# Dense captioning and multidimensional evaluations for indoor robotic scenes

**DOI:** 10.3389/fnbot.2023.1280501

**Published:** 2023-11-14

**Authors:** Hua Wang, Wenshuai Wang, Wenhao Li, Hong Liu

**Affiliations:** ^1^Key Laboratory of Machine Perception, Shenzhen Graduate School, Peking University, Shenzhen, China; ^2^School of Artificial Intelligence, Hebei University of Technology, Tianjin, China

**Keywords:** indoor robotic scene, dense captioning, RGBD fusion, multidimensional evaluation, top-down attention

## Abstract

The field of human-computer interaction is expanding, especially within the domain of intelligent technologies. Scene understanding, which entails the generation of advanced semantic descriptions from scene content, is crucial for effective interaction. Despite its importance, it remains a significant challenge. This study introduces RGBD2Cap, an innovative method that uses RGBD images for scene semantic description. We utilize a multimodal fusion module to integrate RGB and Depth information for extracting multi-level features. And the method also incorporates target detection and region proposal network and a top-down attention LSTM network to generate semantic descriptions. The experimental data are derived from the ScanRefer indoor scene dataset, with RGB and depth images rendered from ScanNet's 3D scene serving as the model's input. The method outperforms the DenseCap network in several metrics, including BLEU, CIDEr, and METEOR. Ablation studies have confirmed the essential role of the RGBD fusion module in the method's success. Furthermore, the practical applicability of our method was verified within the AI2-THOR embodied intelligence experimental environment, showcasing its reliability.

## 1 Introduction

As artificial intelligence technology continues to evolve, mobile robots are taking on increasingly pivotal roles across a multitude of fields (Rubio et al., 2019; Huang et al., 2020; Liu et al., 2022). To enable these robots to more effectively comprehend and adapt to complex, ever-changing indoor environments, it becomes essential to provide a detailed description of the scene (Johnson et al., 2016; Chen et al., 2021). This involves extracting semantic information—such as objects, attributes, and relationships within the scene—and articulating it in natural language. By doing so, we can significantly enhance a robot's perceptual and interactive capabilities, thereby elevating its level of intelligence and the overall user experience (Sheridan, 2016). The task of providing semantic descriptions of scenes is of paramount importance, as it is key to facilitating effective interaction between robots and humans, and crucial to a robot's understanding of human needs.

Scene description refers to the ability of machines to generate high-level natural language descriptions based on given scene images. Several Scene Description methods have been developed for indoor scenes, with a recent focus on Dense Captioning based on 3D point clouds. In 2021, Chen et al. (2021) proposed an end-to-end method called Scan2Cap, which effectively locates and describes 3D objects in the ScanRefer dataset and extracts spatial relationships within the scene. Yuan et al. (2022) introduced a cross-modal Transformer model, X-Trans2Cap, which integrates features from auxiliary 2D modalities into point clouds through knowledge distillation, achieving great performance improvement in this task. Jiao et al. (2022) proposed a multi-level relationship mining model called MORE, aiming to improve 3D Dense Captioning by capturing and utilizing complex relationships within 3D scenes.

The task of providing dense scene captioning presents numerous challenges (Cai et al., 2022). To begin with, in the context of 2D scene captioning, the input from a single modality is often insufficient, making it difficult to discern when objects are occluded or when the viewpoint within the scene changes. Additionally, while 3D scene captioning can capture comprehensive scene information, the computational cost of performing convolution and attention operations on point cloud data is high, and there is an abundance of sparse, irrelevant information. Ultimately, the existing methods of RGBD input have not effectively utilized the information available in depth images, which serves as the motivation for this research. We want to implement a method that could reduce the amount of computation while expressing spatial relationships better, so we came up with RGBD2Cap.

The main contribution of this paper includes the following three aspects: Firstly, we propose a feature extraction method based on RGB+D image multimodal fusion. This method, which is grounded in the transformation between 3D point clouds and 2D images, is combined with a semantic captioning generation module to form RGBD2Cap. Secondly, we design and implement a multi-dimensional evaluation method for scene semantic captioning. This includes both manual and automatic evaluations, and utilizes simulation scenes to assess the model within an embodied intelligence experimental environment. Lastly, the model presented in this article has achieved the highest accuracy according to our evaluation metrics.

## 2 Related work

### 2.1. 2D image and scene captioning

Since its introduction by Johnson et al. (2016), dense captioning has emerged as a subfield of image captioning, with the encoder-decoder architecture becoming the prevailing solution (Cho et al., 2014).

Initial approaches (Mao et al., 2014) to dense image captioning using the encoder-decoder architecture combined Convolutional Neural Networks (CNNs) (LeCun et al., 2015) and Long Short-Term Memory (LSTM) networks (Xu et al., 2015). These methods used the image feature vector extracted by the CNN as the LSTM's initial state and generated descriptive statements word by word.

With the rise of attention mechanisms in natural language processing, methods (Xu et al., 2015; Anderson et al., 2018) combining CNNs and attention mechanisms have emerged. These methods dynamically select the most relevant region feature vectors at each time step based on the current generation state, combining them with global feature vectors as input to subsequent language generation models such as LSTM or Transformer.

Yang et al. (2017) introduced a method that combines joint inference and contextual information fusion to address two significant challenges in the current image-intensive description task. This approach generates improved descriptions by emphasizing visual cues from surrounding salient image regions as contextual features. Kim et al. (2019) introduced a new task, “Relation Captioning,” which generates multiple captions for relational information between objects in an image. They utilized a multi-task triple stream network (MTTSNet) that captures the relational information between detected objects, providing precise concepts and rich representations.

### 2.2. 3D scene captioning

3D vision has become increasingly popular in recent years (Qi et al., 2017; Li et al., 2022; Shao et al., 2022), and 3D detection methods performed on point clouds are becoming more common in 3D vision research.

Chen et al. (2021) pioneered the task of dense captioning in RGB-D scans, a field that has yet to fully explore the discriminative description of objects in complex 3D environments.

In the spirit of neural machine translation, Wang et al. (2022) proposed SpaCap3D. This model features a spatiality-guided encoder and an object-centric decoder, both of which contribute to the generation of precise and spatially-enhanced object captions.

However, existing methods often overlooking contextual information such as non-object details and background environments within point clouds. To address this, Zhong et al. (2022) utilized point cloud clustering features as contextual information, incorporating non-object details and background environments into the 3D dense captioning task.

Jiao et al. (2022) aimed to improve 3D dense captioning by capturing and utilizing complex relations within the 3D scene. They proposed MORE, a Multi-Order RElation mining model, to generate more descriptive and comprehensive captions. Chen et al. (2022) introduced UniT3D, a fully unified transformer-based architecture for jointly solving 3D visual grounding and dense captioning.

Although the representation of 3D point cloud scenes has achieved considerable performance to some extent, its computational overhead remains excessively large. This is primarily due to the sparsity of the 3D point cloud information, which impedes the efficient utilization of features. This paper proposes a method based on RGBD static images, effectively integrating RGB and Depth features. While reducing computational load, this approach also ensures the model's acquisition of spatial information, thereby enhancing the accuracy of the generated descriptions.

## 3 Proposed method

**Figure 1:**
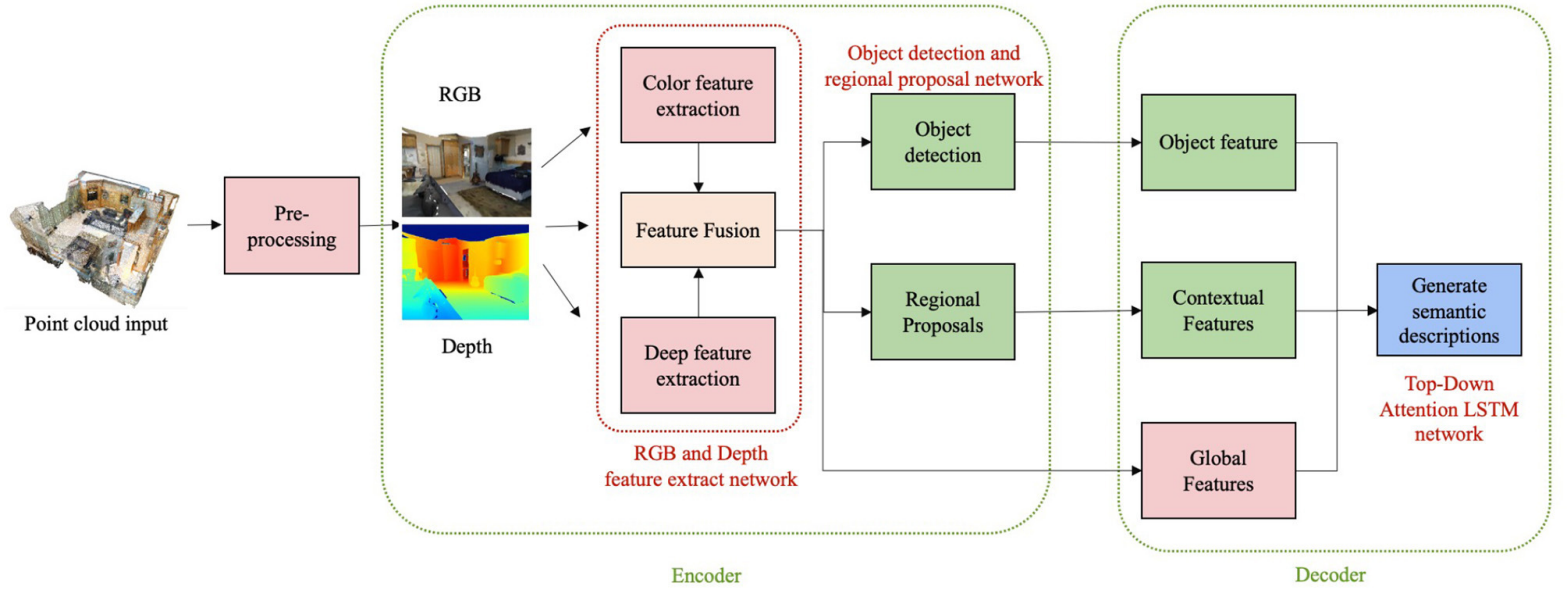
The general structure of the proposed method.

The research of this paper is to train a deep learning model based on the RGBD images corresponding to indoor 3D scenes, so that it can automatically generate the corresponding linguistic descriptions. In order to accomplish these goals, this paper accomplish the following specific tasks. First, we need to pre-process the original point cloud data to obtain 2D and depth images corresponding to different objects in the scene. Then, we design a RGB and Depth multimodal feature extraction network to extract and fuse the features of RGB and depth images. In addition, we need a target detection network to detect the objects in the scene images so that the subsequent Top-down Attention LSTM model can accurately understand the objects in the images. Finally, the features extracted by the neural network are fed into the text generation network to generate text for the purpose of understanding the high-level semantic information of the scene. The overall structure of the proposed method is shown in Figure 1.

### 3.1. Rendering of 3D scenes

This study employs the ScanRefer (Chen et al., 2020) dataset for model training, which is an extension of the ScanNet dataset with added high-level semantic descriptions. ScanNet provides a rich array of indoor 3D scene meshes, semantic labels, and 2D video frame images with corresponding depth maps. However, we refrain from using ScanNet's 2D image data directly for training due to the blurriness of most images, which hampers effective capture of the scene's visual information. Instead, we use the viewpoints provided by the ScanRefer dataset to render the 3D data, yielding clearer 2D data.

The rendering process of the 3D scene adheres to the principle of camera projection (Kannala and Brandt, 2006). It begins with transforming the scene points in the world coordinate system using the camera's external parameter matrix, yielding their coordinates in the camera's coordinate system. These points are then converted to the image coordinate system using the camera's internal parameter matrix.

The initial step involves the transformation from the world coordinate system to the camera coordinate system, a rigid transformation composed of translation and rotation. In this study, a right-hand coordinate system is used for world coordinates. If a point in the scene has coordinates (*x, y, z*) in the world coordinate system. We aim to obtain its coordinates (*x*′, *y*′, *z*^′)^ in the camera coordinate system, this can be achieved through the following matrix transformation:


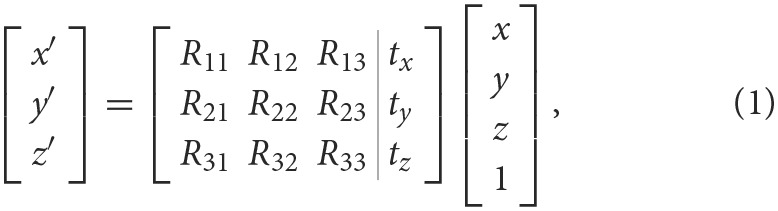


where *t* represents the translation vector of the point coordinates, and the orthogonal matrix *R* represents the rotation matrix of the point's coordinates in space. The values of both are determined by the position of the camera in the world coordinate system and the direction of the optical axis. The external parameter matrix of the camera is composed of the rotation matrix *R* and the translation vector *t*, represented as [*R*|*t*]∈*R*^3 × 4^.

Next is the transformation from the camera coordinate system to the normalized device coordinate system, which is usually achieved through perspective projection. For a point (*x*′, *y*′, *z*^′)^ in the camera coordinate system, the following matrix transformation can be used to describe this process:


(2) $$\left[\begin{array}{c} x^{\prime \prime }\\ y^{\prime \prime }\\ z^{\prime \prime } \end{array}\right]\begin{array}{l} =\left[\begin{array}{cccc} f & 0 & 0 & 0\\ 0 & f & 0 & 0\\ 0 & 0 & 1 & 0 \end{array}\right]\left[\begin{array}{c} x^{\prime }\\ y^{\prime }\\ z^{\prime }\\ 1 \end{array}\right]\text{,} \end{array}$$

where *f* represents the focal length of the camera, and (*x*^′′^, *y*^′′^, *z*^′′)^ are the coordinates of the point in the normalized device coordinate system. This transformation maps the 3D points in the camera coordinate system to a 2D, while preserving the depth information of each point.

Finally, there is the transformation from the normalized device coordinate system to the image coordinate system, which can be achieved through the simple scaling and offset. For a point (*x*^′′^, *y*^′′^, *z*^′′)^ in the normalized device coordinate system, we want to obtain its coordinates (*u, v*) in the image coordinate system, which can be achieved through the following formula:


(3) $$\left[\begin{array}{c} u\\ v \end{array}\right]\begin{array}{l} =\left[\begin{array}{cc} w/2 & 0\\ 0 & h/2 \end{array}\right]\left[x^{\prime \prime }y^{\prime \prime }\right]+\left[\begin{array}{c} w/2\\ h/2 \end{array}\right]\text{,} \end{array}$$

where *w* and *h* represent the width and height of the image, respectively. This transformation maps the points in the normalized device coordinate system to the image coordinate system, generating the final 2D image.

The above is the whole process we used to convert the point cloud in the scene, from the world coordinate system to the image coordinate system. The whole process is linear and can be achieved by a series of matrix multiplications. This allows us to obtain a mapping of the 3D point cloud data onto the 2D image, which can then be processed and analyzed using 2D image processing techniques.

### 3.2. RGB and depth multimodal fusion networks

The network accepts an RGB image and a depth image as inputs. Its architecture is grounded in ResNet101 (He et al., 2015), a deep residual network of 101 convolutional neural network layers. This network addresses the issues of vanishing and exploding gradients, common in deep neural network training, through residual learning.

**Figure 2:**
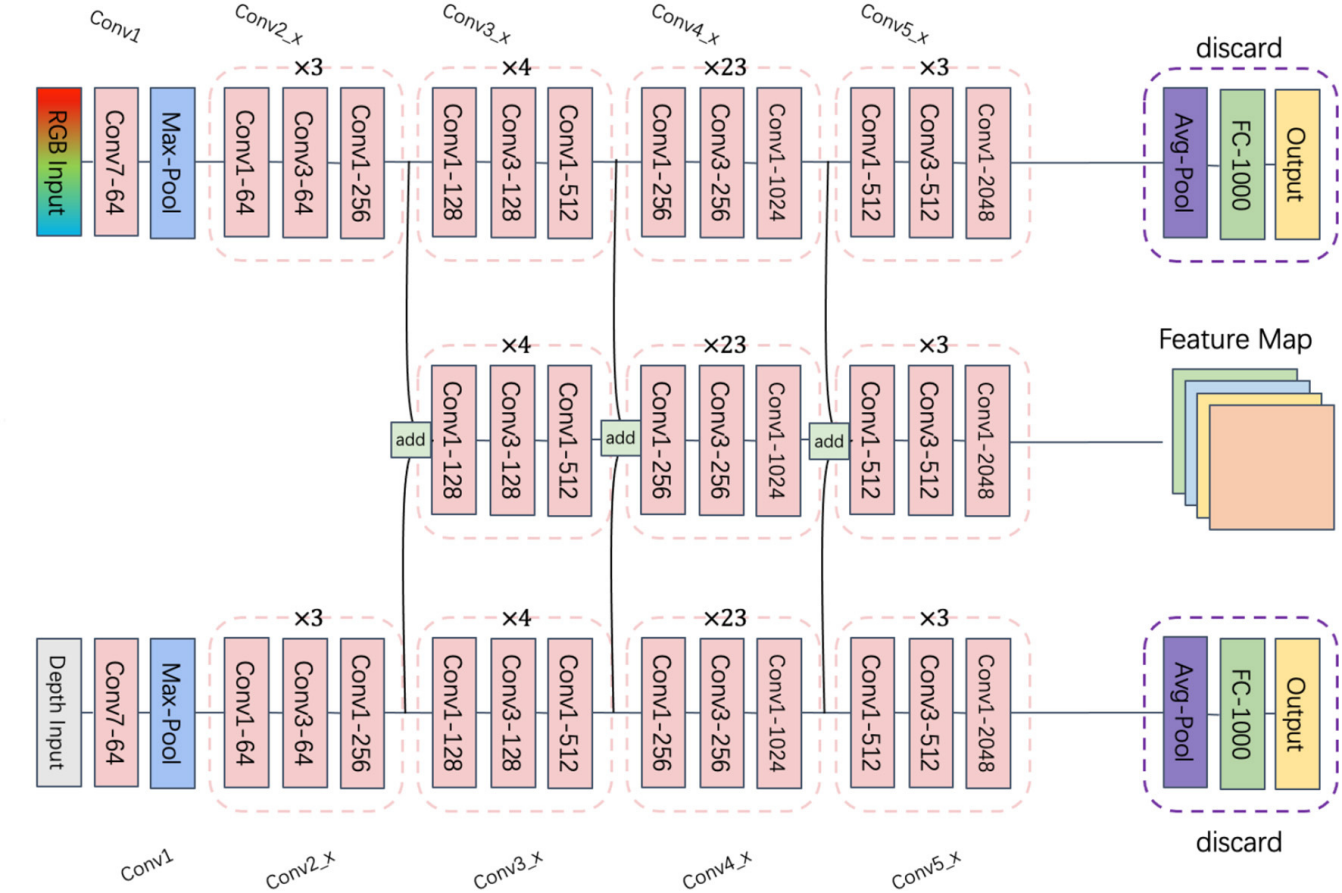
RGB and Depth multimodal fusion networks.

The feature fusion approach employed in this network is a third-branch multilevel fusion, as shown in Figure 2. Specifically, we start with the feature map generated by the third convolutional layer of ResNet101. The RGB and depth feature maps from this convolutional layer are summed and fused separately to form the network's third branch. The same convolutional operation is performed on this third branch, and the feature maps obtained from subsequent convolutional layers are continuously added to yield the final RGBD multimodal features.

Our feature extraction network is bifurcated into two branches: the RGB branch and the depth branch. The RGB image and the depth image are processed through their respective convolution layers to extract features and generate their individual feature maps. These two feature maps are then fused using the feature fusion method to obtain RGBD multimodal features, which serve as the third branch for multilevel fusion. This network omits the final fully-connected and softmax layers of ResNet, bypassing classification result output and directly utilizing its feature maps for subsequent tasks.

### 3.3. Target detection and region proposal network

The Bottom-Up and Top-Down Attention model (Anderson et al., 2018) comprises two components: a bottom-up image feature extractor and a top-down language generator. The bottom-up image feature extractor employs a Faster-RCNN (Ren et al., 2015) detector to identify a set of potential visual regions, generating a fixed-length feature vector for each region.

**Figure 3:**
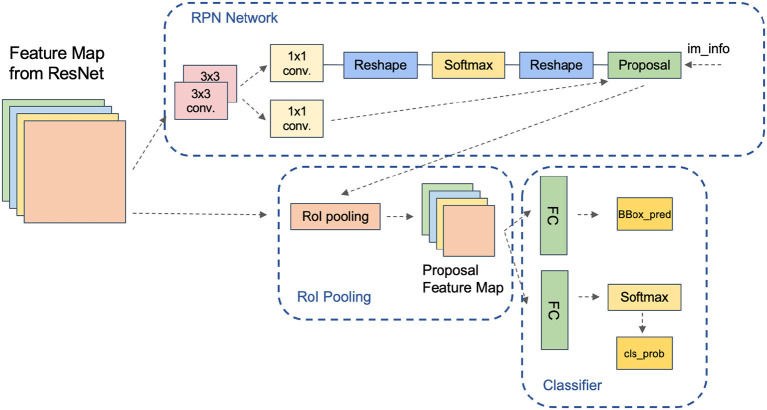
Target detection and region proposal network.

As shown in Figure 3, this study employs a Faster-RCNN-based object detection and region proposal network, utilizing the previously mentioned multimodal fusion ResNet101 as its backbone, augmented with an RPN network and an RoI Pooling layer. The RPN network, which is fully convolutional, generates candidate bounding boxes. It takes the output feature map of the backbone network as input and produces a series of candidate bounding boxes along with their corresponding scores. A 3 × 3 convolution generates scores for each position, and non-maximum suppression is applied to eliminate overlapping candidate boxes. The RoI Pooling layer takes the output feature map of the backbone network and a series of candidate boxes as input, outputting a fixed-size feature vector after pooling. The final pooling results are concatenated to form the ultimate feature vector.

### 3.4. Top-down attention LSTM network

**Figure 4:**
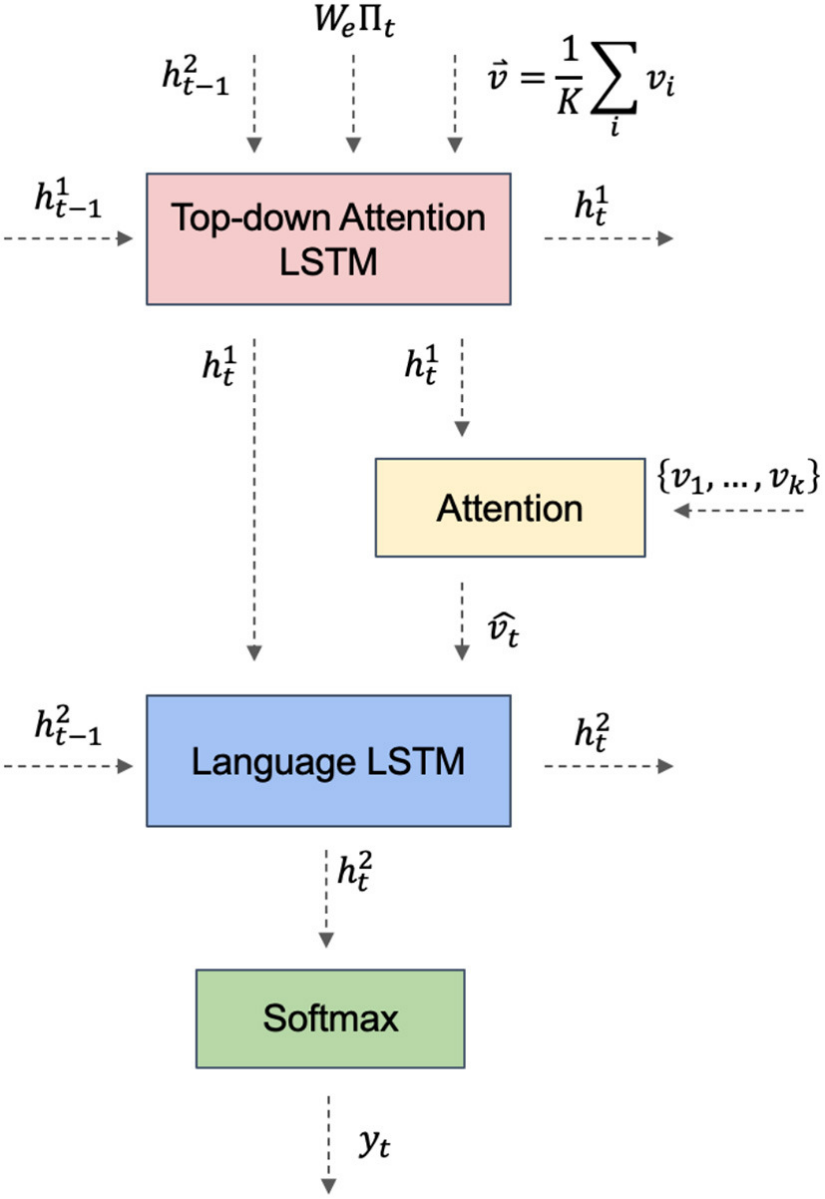
Top-down attention LSTM network.

The top-down language generator in the Bottom-Up and Top-Down Attention model employs an attention mechanism as shown in Figure 4. This mechanism uses the currently generated word as a query, calculates its similarity with the bottom-up feature vector, and produces a set of attention weights. These weights are then used to compute a weighted average of each feature vector, which is used to generate the next word.

The top-down attention mechanism is the heart of the model. The model uses the currently generated word as a query at each time step, calculates its similarity with the bottom-up feature vector, and produces a set of attention weights. These weights are then used to compute a weighted average of each feature vector, which is used to generate the next word. This attention mechanism can be viewed as a top-down interpretation of the image, integrating the generated language with the underlying image representation to produce a more precise image description.

In this module, the global features, object features, and context features obtained from the previous networks are fused, and to utilize these three features effectively, we use the following method for fusion. Firstly, the global and target features with the same dimension are spliced and fused, then a fully connected network with an activation function is used to scale the fused features to the same dimension as the contextual features, and then they are spliced twice to get the final fused features, which can effectively utilize the extracted contextual features.

## 4 Experiments

### 4.1. Dataset

Generating scene descriptions for robots necessitates a computer vision approach that can convert environmental data into natural language descriptions. Several datasets have been developed to provide high-level language descriptions for various scenes, including the ScanRefer dataset.

ScanRefer (Chen et al., 2020) is a dataset designed explicitly for dense scene descriptions, primarily used in robotic indoor scene understanding tasks. It provides semantic scene description information, facilitating robots' comprehension of their surroundings. The dataset comprises 800 annotated scenes, 11,046 stereo location frames of objects, and 51,583 corresponding textual descriptions. It offers not only a wealth of scene description data but also high-quality 3D scene data. By employing 3D projection, we can map the objects in the scene onto a 2D plane, making it suitable for the RGBD2Cap model presented.

**Figure 5:**
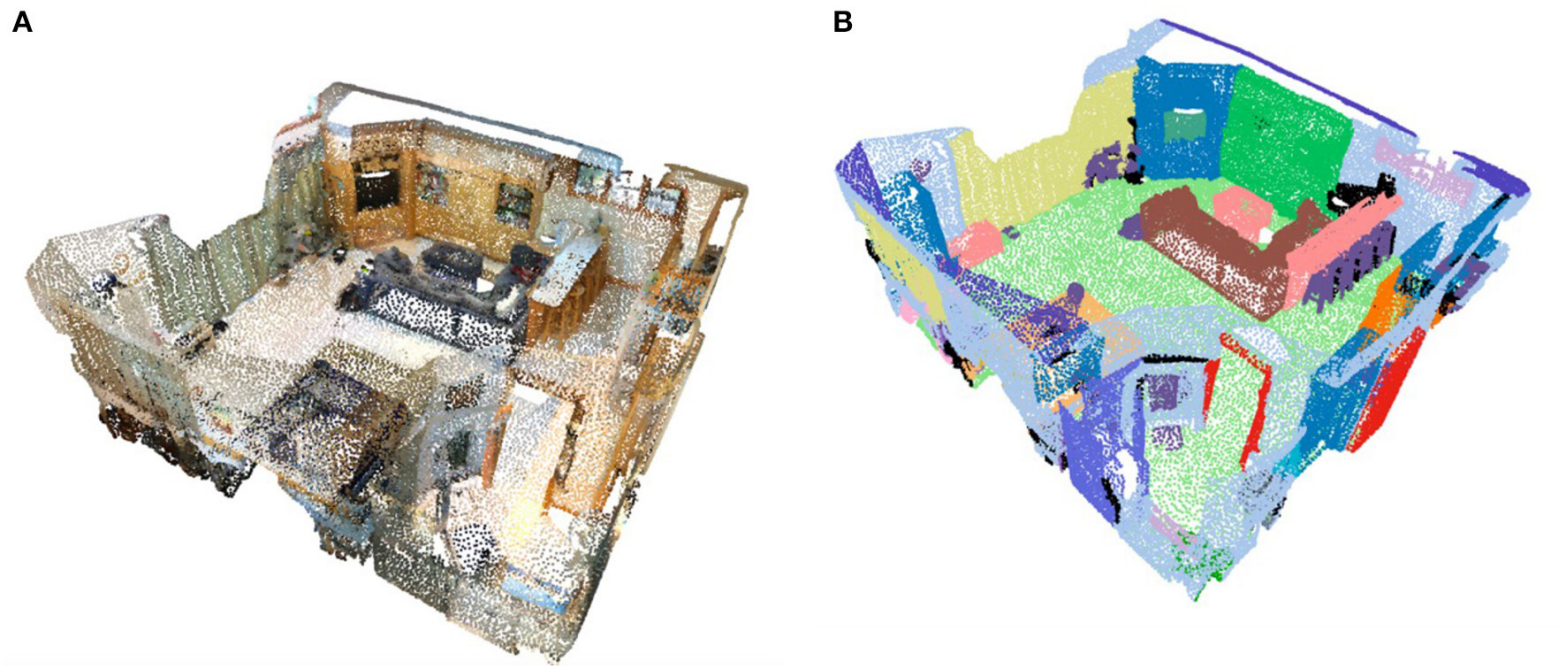
**(A)** Visualization of point cloud data. **(B)** Visualization of the labels of point cloud data.

ScanRefer builds upon the ScanNet (Dai et al., 2017) dataset by adding natural language descriptions. As shown in Figures 5A, B, ScanNet provides 3D point clouds and their corresponding semantic labels, resulting from high-quality scene reconstruction. In this study, we utilize the 3D data from the dataset and select viewpoints provided by ScanRefer to render the point cloud scenes. The authors of ScanRefer provide viewpoint information for different camera locations in each scene in the Annotated viewpoints file. This information includes the camera location, rotation angle, and look at (the point the camera is currently aimed at), which we use to set the camera pose.

### 4.2. Rendering of 2D images

**Figure 6:**
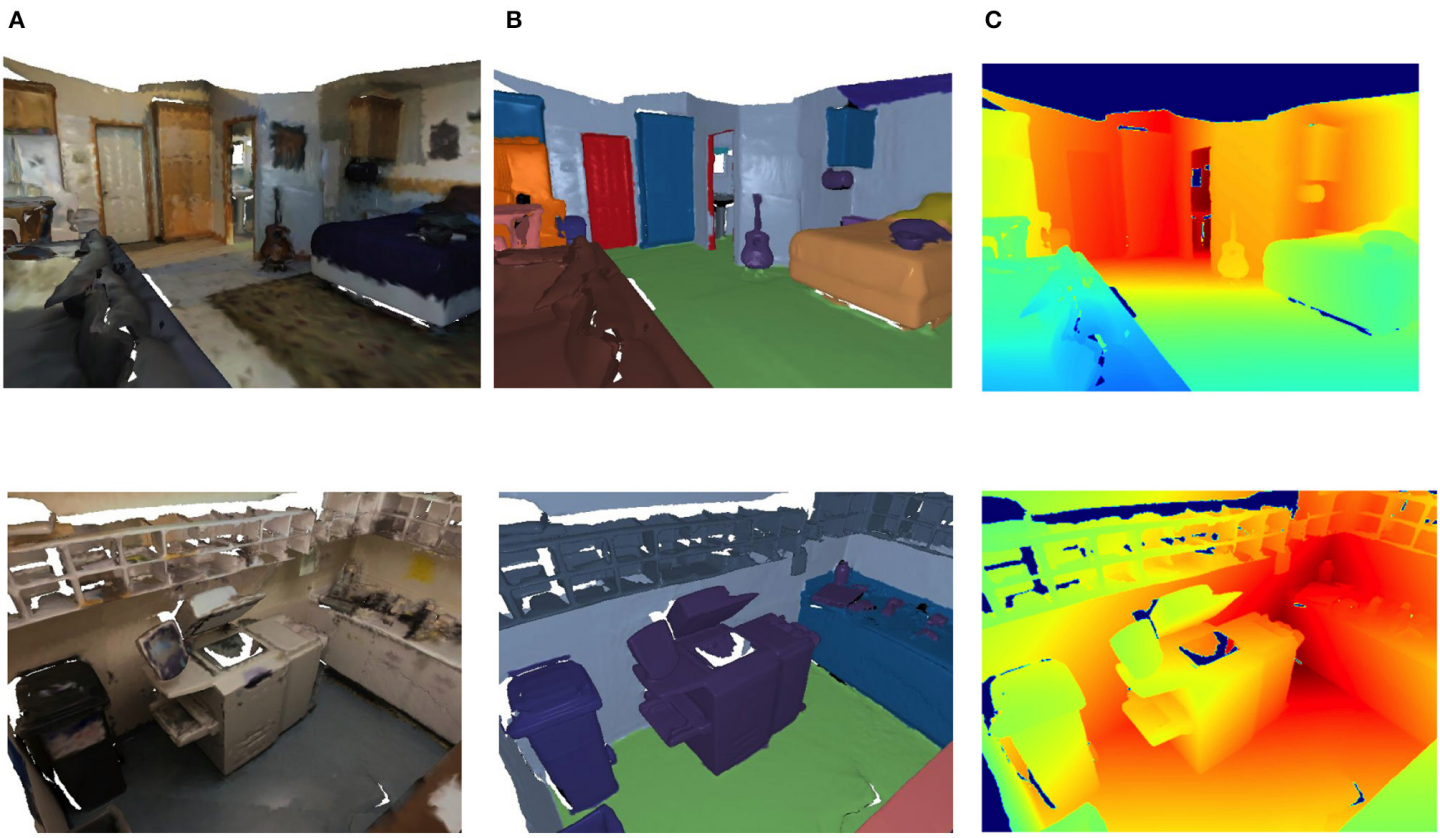
Multi-view image based on pytorch3d rendering. **(A)** RGB image. **(B)** Labeled image. **(C)** Depth image.

The rendering of the 3D scene using Pytorch3D (Ravi et al., 2020) is shown in Figure 6. From left to right, the RGB color image of a viewpoint, the rendered image with labels, and the depth image are shown.

### 4.3. Configuration of the training model

This study utilized the Python programming language and the PyTorch deep learning framework to implement the algorithm. The hardware setup for the experiment included a NIVIDA Tesla P100 GPU (16GB), 80GB of RAM, and 70GB of available disk space. The software environment was configured with Ubuntu 18.04, Python 3.8, Cuda 11.1, and PyTorch 1.8.1.

The experimental procedure began with the fusion of the ScanRefer dataset with RGBD images to extract image features. The primary architecture used in the training process was a convolutional neural network and a long short-term memory network. The model was trained using the Adam optimizer, with a batch size of 14 and 100 epochs. The initial learning rate was set at 0.0005, and a weight decay parameter of 0.0001 was used to control model complexity. Intersection over Union (IOU) thresholds were set at 0, 0.25, and 0.5. The number of sampled point clouds was 40,000, with 562 scenes in the training set and 141 in the validation set. After rendering, the training set comprised 36,665 samples, and the validation set included 9,508 samples.

The loss of the RGBD2Cap network is a multi-task loss, including target detection loss and semantic description loss. The loss for target detection includes classification loss and bounding box regression loss, while the text generation part can directly use the cross-entropy loss of text prediction probability. The final multi-task loss value at the end of model training was 0.26.

### 4.4. Scene dense captioning and evaluation methods

#### 4.4.1. Metrics-based evaluation

The objective of the dense captioning task is to identify and articulate all objects and events of interest within an image. This task merges two subtasks: object detection and image captioning. Consequently, its evaluation metrics are a fusion of the metrics used for these two subtasks.

Firstly, the Mean Average Precision (mAP) is typically used as the evaluation metric for object detection. The mAP represents the Area Under Curve (AUC) of the average precision-recall curve across all categories. For each category, detections are ranked based on their predicted confidence, followed by the calculation of precision and recall. The precision-recall curve is then plotted, and the area under it is calculated to obtain that category's Average Precision (AP). The final mAP is obtained by averaging the AP across all categories.

Secondly, image captioning is evaluated using metrics such as BLEU, CIDEr, Meteor, and Rouge. BLEU (Bilingual Evaluation Understudy) (Papineni et al., 2002) assesses the similarity between generated and reference descriptions primarily through *n*-gram accuracy. CIDEr (Consensus-based Image Description Evaluation) (Vedantam et al., 2015) gauges the quality of descriptions by calculating the TF-IDF-weighted cosine similarity between generated descriptions and a set of reference descriptions. Meteor (Metric for Evaluation of Translation with Explicit ORdering) (Banerjee and Lavie, 2005) and Rouge (Recall-Oriented Understudy for Gisting Evaluation) (Lin, 2004) evaluate description quality by computing the longest common subsequence between generated and reference descriptions.

In dense captioning tasks, these evaluation metrics for object detection and image captioning are typically used in conjunction. Specifically, mAP is used to assess the model's performance on the object detection task, while BLEU, CIDEr, Meteor, and Rouge are used to evaluate the model's performance on the image captioning task. Finally, these evaluation metrics can be combined in a weighted manner to derive a comprehensive evaluation metric for assessing the model's overall performance on the dense captioning task.

In this paper, we evaluate the completed training model and obtain several evaluation metrics data, including (BLEU1-4, cider, mAP@0.5, meteor, rouge, and many other evaluation metrics). The IOU thresholds *k* in the data table are all taken as 0.5. The results are shown in Table 1.

**Table 1:** Algorithm comparison and ablation study on RGBD2Cap components.

	**BLEU-4**	**CIDEr**	**ROUGE-L**	**METEOR**
RGB (DenseCap) (Johnson et al., 2016)	20.1	32.7	38.2	21.0
RGB (without fusion)	20.7	34.5	41.6	22.9
Show and tell (without attention)	18.3	33.5	**46.9**	21.09
RGB+D fusion (ours)	**21.5**	**35.1**	38.8	**23.3**

Bold values indicate the optimal value of each method for a given evaluate metric.

Since no experimental studies are based on RGBD fusion so far, the proposed model is compared with the algorithm without RGBD fusion.

The RGB(DenseCap) row in Table 1 uses the rendered RGB image as input, and the Dense Captioning of the scene is obtained by using the method in paper[]. The RGB(Without Fusion) line also takes the same image as input and uses the RGBD2Cap network without the Depth branch and the third branches to get the DenseCap. The last row in Table 1 is our complete proposed RGBD2Cap method. Based on the data in the table, it can be seen that the performance of the proposed model is optimal in the three indexes of BLEU-4, CIDEr, and METEOR, which can verify the effectiveness of the RGBD fusion module.

Furthermore, ablation experiments were conducted to ascertain the effectiveness of the Top-down Attention and FasterRCNN modules. As depicted in Table 1, the model's performance across all three metrics declines when the Attention module is not utilized, indicating the module's crucial role in feature extraction during semantic description generation.

In addition, we compare the proposed method RGBD2Cap with the 3D method Scan2Cap (Chen et al., 2021), and the obtained results are shown in Table 2. Both methods are trained on the ScanRefer dataset, the difference is that RGBD2Cap uses a rendered RGBD image as the input to the model, while Scan2Cap directly uses a 3D point cloud as the input. Both models are trained on a 2080Ti GPU for 50 epochs to ensure fairness. Based on the experimental results, it can be learned that although the 3D model outperforms our method in the three metrics, its training time is 9 times longer than that of RGBD2Cap, greatly shortening the training time while reducing the performance loss.

**Table 2:** Comparison of time and accuracy between 2D and 3D methods.

	**BLEU-4**	**CIDEr**	**ROUGE-L**	**METEOR**	**Train time (h)**
RGBD2Cap (ours)	21.5	35.1	38.8	**23.3**	**8**
Scan2Cap (Chen et al., 2021)	**23.32**	**39.08**	**44.78**	21.97	71

Bold values indicate the optimal value of each method for a given evaluate metric.

Lastly, we verify the impact of the Faster-RCNN module's detection capabilities on the description performance by contrasting it with the actual bounding box, as shown in Table 3. The features extracted using the real bounding box of the object are more precise, hence the semantic description based on it will also yield more accurate descriptions. Following experimental verification, it was found that the model exhibits a slight decrease in the four indicators. Still, the decrease is minimal, thus affirming the feasibility of the end-to-end model. The target features produced using Faster-RCNN as the target detector and feature box extractor serve as a solid foundation for semantic description.

**Table 3:** Performance of using Faster-RCNN as a target detector vs. real bounding box to generate description results.

	**BLEU-4**	**CIDEr**	**ROUGE-L**	**METEOR**
Faster-RCNN	21.5	35.1	38.8	23.3
Ground truth	**24.3**	**35.7**	**39.3**	**23.5**

Bold values indicate the optimal value of each method for a given evaluate metric.

#### 4.4.2. Manual evaluation

Because the high-level semantics are more difficult to describe formalistically, manual evaluation is essential, and this paper next evaluates a manual sample of training results.

**Figure 7:**
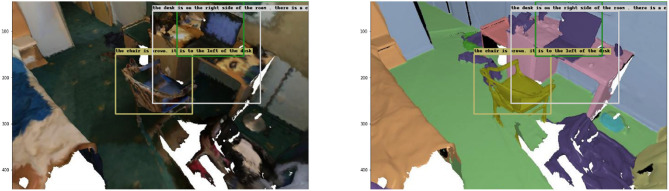
Example 1 of dense captioning results in the validation set.

A randomly selected sample from the validation set was used for inference prediction, and the results are presented in Figure 7. The captioning of the red box is “*The chair is brown. It is to the left of the desk”*, in which the object's color information and spatial location are accurately displayed; the captioning of the white box is “*The monitor is on the desk on the right side. It is the monitor that is closest to the window”*, although the real label of the computer on the desktop is “laptop”, the object vocabulary “monitor” given in the description is similar; this description shows very detailed spatial location information; the captioning of the green box is “*The desk is on the right side of the room. There is a chair in front of the desk.”* This description shows the position of the desk object in the room and accurately expresses its spatial relationship with the chair in front of it.

**Figure 8:**
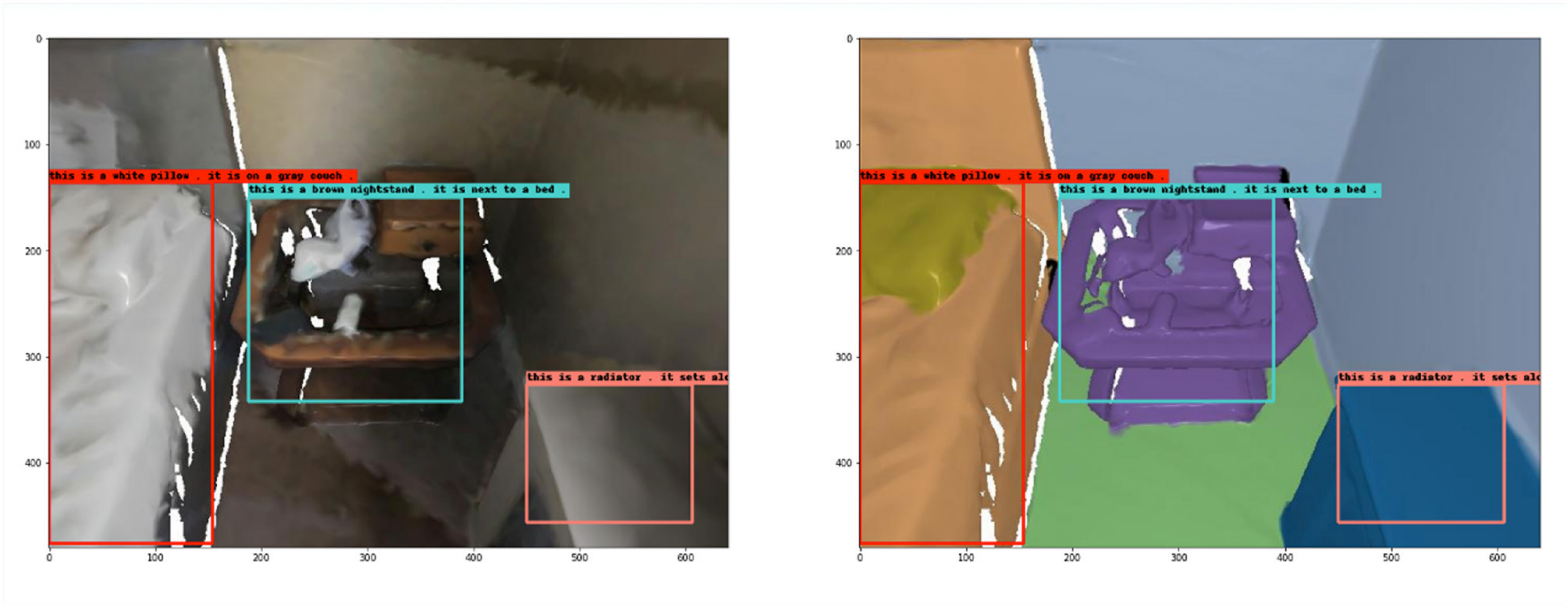
Example 2 of dense captioning results in the validation set.

However, not all scenes are accurately described, and Figure 8 shows another randomly selected sample from the validation set. The captioning of the red box in the figure is “*This is a white pillow. It is on a gray couch.”* Although the object's color is accurately described as white, the white bed sheet is mistakenly identified as a pillow and the bed below as a sofa, which is a misjudgment. The text of the blue box is “*This is a brown nightstand. It is next to a bed”*, which accurately shows that the object is a brown nightstand; it also points out that its orientation is next to the bed; the text of the pink box is “*this is a radiator. It sets along the wall.”* This sentence incorrectly identifies the object as a radiator, probably because the picture shows an incomplete object, but it correctly conveys that the object is against the wall.

From the results, it can be seen that the current field still faces many challenges, and future research directions could be more fine-grained feature extraction to achieve a more accurate description.

### 4.5. Simulation tests in AI2-THOR

#### 4.5.1. AI2-THOR

AI2-THOR is an embodied AI experimental environment designed to simulate real-world environments to train and test AI systems (Kolve et al., 2017; Deitke et al., 2020). This simulation environment contains a variety of detailed indoor scenarios such as kitchen, bedroom, bathroom, and living room. In AI2-THOR, AI intelligence can explore and interact with the environment through a series of actions, such as moving, viewing, grasping, and manipulating objects. This design allows the intelligent body to learn and understand the properties and relationships of objects in the environment and how they affect the execution of tasks as it performs them.

A key feature of AI2-THOR is its support for scene semantics, for which objects are provided with labels with semantic information. In this paper, RGBD2Cap is further evaluated by controlling the actions of the intelligence in AI2-THOR, acquiring single frames of images in the scene and their depth images as input samples for the model, and observing the correlation between the model's output and the images.

#### 4.5.2. Operation details

The operation of AI2-THOR is facilitated through Python, with the research team providing a Python API for public experimentation. Initially, the AI2-THOR experimental environment is installed and initialized, typically involving the selection of a scene (e.g., kitchen, bedroom, etc.) and establishing the AI agent's initial position and orientation. Once the environment is initialized, the agent is primed to commence action execution.

The system's “move” and “rotate” actions can be utilized to capture a single frame from varying scene perspectives. For instance, the AI agent can be maneuvered forward, backward, or rotated left or right. Each execution of these actions provides the agent with a new viewpoint for frame acquisition. To procure a depth image, the “Get Depth Image” function of the AI2-THOR environment is employed, returning a depth image that represents the scene's depth from the AI system's current viewpoint. The depth image is a two-dimensional array, with each element representing the depth value of the corresponding pixel. These depth values serve to comprehend the position and shape of objects within the scene.

**Figure 9:**
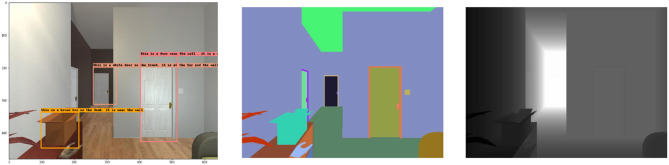
RGB, Labeled, and Depth images of scenes in AI2-THOR environment.

The paper randomly selects a scene in the experimental environment, and after initializing the intelligent body in the scene, the movement method and the final location and angle were arbitrarily set, and the RGB, Depth and instance labeled images of the scene were captured. The effect of the model was verified, and the results are shown in Figure 9. The text corresponding to the three detection boxes are “*This is a white door in the front. it is at the far end of the wall.”*, “*This is a brown box on the desk. It is near the wall. It is near the wall.”*, “*This is a door near the wall. It is a white door.”* It can be seen that these description results are relatively accurate, and the model has excellent performance in the test results in the simulation environment.

## 5 Conclusion

In this paper, the problem of scene semantic description for indoor mobile robots is studied. The ScanNet scene data is processed to obtain its RGBD image, and then the corresponding semantic description is obtained based on the RGBD image. After experiments, we know that the proposed algorithm can effectively describe the indoor scene semantically. The use of multimodal information can help the model understand the scene better and improve the accuracy of the model. Compared with direct RGB image recognition, the proposed model obtains better results in three indexes, such as BLEU, CIDEr, and METEOR, and gets better test performance in the AI2-THOR experimental environment. Overall, the proposed method has high practicality and promotion value and can provide more accurate and advanced semantic information for the perception of indoor mobile robots.

## Data availability statement

The raw data supporting the conclusions of this article will be made available by the authors, without undue reservation.

## Author contributions

HW: Investigation, Methodology, Writing—original draft. WW: Conceptualization, Data curation, Supervision, Writing—original draft. WL: Data curation, Supervision, Writing—review & editing. HL: Funding acquisition, Supervision, Writing—review & editing.
